# SNP-based pathway enrichment analysis for genome-wide association studies

**DOI:** 10.1186/1471-2105-12-99

**Published:** 2011-04-15

**Authors:** Lingjie Weng, Fabio Macciardi, Aravind Subramanian, Guia Guffanti, Steven G Potkin, Zhaoxia Yu, Xiaohui Xie

**Affiliations:** 1 Department of Computer Science, University of California, Irvine, CA, USA; 2 Department of Psychiatry & Human Behaviour, University of California, Irvine, CA, USA; 3 Broad Institute of MIT and Harvard, Cambridge, MA, USA; 4 Department of Statistics, University of California, Irvine, CA, USA; 5 Institute for Genomics and Bioinformatics, University of California, Irvine, CA, USA

## Abstract

**Background:**

Recently we have witnessed a surge of interest in using genome-wide association studies (GWAS) to discover the genetic basis of complex diseases. Many genetic variations, mostly in the form of single nucleotide polymorphisms (SNPs), have been identified in a wide spectrum of diseases, including diabetes, cancer, and psychiatric diseases. A common theme arising from these studies is that the genetic variations discovered by GWAS can only explain a small fraction of the genetic risks associated with the complex diseases. New strategies and statistical approaches are needed to address this lack of explanation. One such approach is the pathway analysis, which considers the genetic variations underlying a biological pathway, rather than separately as in the traditional GWAS studies. A critical challenge in the pathway analysis is how to combine evidences of association over multiple SNPs within a gene and multiple genes within a pathway. Most current methods choose the most significant SNP from each gene as a representative, ignoring the joint action of multiple SNPs within a gene. This approach leads to preferential identification of genes with a greater number of SNPs.

**Results:**

We describe a SNP-based pathway enrichment method for GWAS studies. The method consists of the following two main steps: 1) for a given pathway, using an adaptive truncated product statistic to identify all representative (potentially more than one) SNPs of each gene, calculating the average number of representative SNPs for the genes, then re-selecting the representative SNPs of genes in the pathway based on this number; and 2) ranking all selected SNPs by the significance of their statistical association with a trait of interest, and testing if the set of SNPs from a particular pathway is significantly enriched with high ranks using a weighted Kolmogorov-Smirnov test. We applied our method to two large genetically distinct GWAS data sets of schizophrenia, one from European-American (EA) and the other from African-American (AA). In the EA data set, we found 22 pathways with nominal P-value less than or equal to 0.001 and corresponding false discovery rate (FDR) less than 5%. In the AA data set, we found 11 pathways by controlling the same nominal P-value and FDR threshold. Interestingly, 8 of these pathways overlap with those found in the EA sample. We have implemented our method in a JAVA software package, called *SNP Set Enrichment Analysis *(SSEA), which contains a user-friendly interface and is freely available at http://cbcl.ics.uci.edu/SSEA.

**Conclusions:**

The SNP-based pathway enrichment method described here offers a new alternative approach for analysing GWAS data. By applying it to schizophrenia GWAS studies, we show that our method is able to identify statistically significant pathways, and importantly, pathways that can be replicated in large genetically distinct samples.

## Background

The power of genome-wide association studies (GWAS) to discover common genetic variants associated with complex traits has been empirically demonstrated [1-6]. The single-SNP analysis tests genetic association on individual SNPs and identifies only the most significant SNPs because of the stringent statistical criteria necessary for minimizing false positive hits. The identified SNPs, however, represent only a small fraction of the genetic variants contributing to complex traits; the majority of the variations remain hidden within the statistical ''noise'' [7,8]. Genetic variants with small individual effect sizes but jointly significant genetic effects would be missed by single-SNP analysis. As a result, identified genetic variants only explain a small fraction of heritability for most studied traits[9].

It is increasingly recognized that pathway-based analysis, which considers cumulative association between the outcome and a group of SNPs or genes in a biological pathway, can greatly complement the single-SNP approach in understanding genetic determinants of common diseases as well as providing insight into the biological process of complex diseases [10-15]. A pathway-based analysis by Baranzini *et al *[16] not only confirmed previously identified immunological pathways but also found that neural pathways might be responsible for multiple sclerosis. Joel Hirschhorn [11] pointed out that for many diseases, different risk loci are often clustered in a common pathway, so when a study highlights the role of one or a group of loci in a disease, it also provides important insights and predictive information on the role of other loci within the same biological group. He argued that the primary goal of genome-wide association studies should not be the prediction of individual risk loci but rather the discovery of biological pathways underlying polygenic diseases and traits. The genetic variants revealed in pathway-based analysis could be used to build predictive models for complex diseases, and provide insights on how multiple genetic variants jointly contribute to the etiology of complex human diseases.

One approach for pathway association analysis of GWAS is to extend the gene set enrichment analysis (GSEA) method, which has been successfully applied in gene expression data analysis [17]. However, a key difference between gene expression analysis and GWAS analysis is that each gene in GWAS is represented by many SNPs. The challenge is to determine the number as well as which SNPs are the best representatives for each gene.

Most of the current methods for pathway analysis of GWAS data are gene-based. Wang *et al*. [13] used the SNP with the strongest association to represent a gene. Choosing the smallest P-value for each gene might not be optimal in situations when the joint action of multiple SNPs within a gene explains more variance than the most significant SNP. For example, if a gene contains multiple causal variants, it might not be identified by the smallest P-value method, which reduces the power of the subsequent pathway enrichment analysis. Moreover, this approach is likely to favour genes of large sizes, as genes with a larger number of SNPs have a higher chance of having significant SNPs, by chance alone. Consequently, the effects of genes with smaller numbers of SNPs would be underestimated. Holmans *et al*. [10] proposed ALIGATOR (Association LIst Go AnnoTatOR) method to study the significance of pathways. Although this method corrects variable gene sizes by simulations, it requires a pre-determined P-value cutoff for selecting significant SNPs and the evaluation of pathways is gene-based, not SNP-based. Yu *et al*. [18] used an adjusted P-value for each gene, and also treated gene as the basic unit for analysis. Since the gene-based approaches focused on testing significance at the gene-level, they may have low power to detect pathways containing only a few genes[19].

Recently, Holden *et al*. [20] proposed a SNP-based pathway analysis, which used all available SNPs to represent a gene. This approach is computationally intensive and might not be practical for genome-wide studies with millions of SNPs. O'Dushlaine *et al*. [21] developed a SNP ratio test (SRT) method which computed the ratio of the number of significant (P < 0.05) to the number of non-significant (P >= 0.05) SNPs for each pathway and used permutations to identify the significant pathways. The SNP ratio test (SRT) method computes the ratio of the number of significant (P < 0.05) to the number of non-significant (P >= 0.05) SNPs for each pathway, and uses permutations to quantify its statistical significance. If there is only one gene that contains significant SNPs, the SRT method would reduce the pathway signal to a gene signal. By contrast, our method uses adaptive rank truncated product and permutations to determine the number of representative SNPs for each gene, and each gene includes at least one SNP. Therefore contributions from multiple genes are emphasized in the pathway analysis. Another disadvantage of the SRT method is that it treats all significant SNPs equally, which can lead to a reduction of power in detecting significant pathways.

To address these limitations, we propose a new SNP-based pathway analysis method, called SNP Set Enrichment Analysis (SSEA), for GWAS studies. SSEA consists of two main steps: selecting representative SNPs for each gene, and performing pathway enrichment analysis using all selected SNPs. In the first step, we exploit an adaptive rank truncated product method with permutations to choose the most significant subset of SNPs for each gene. The number of SNPs representing a gene is not predetermined, but data driven. Then for each pathway, we calculate the average number of representative SNPs for the genes within this pathway and re-select SNPs using this number. In the second step, we modify the existing GSEA algorithm [17] to conduct the pathway enrichment analysis using all selected SNPs. We rank all SNPs selected from the first step based on their strength of association with the trait, and then test whether the set of SNPs associated within a pathway is significantly enriched with high ranks using a weighted Kolmogorov-Smirnov test. Because this test is rank-based, SNPs with smaller P-values tend to contribute more in a pathway.

## Methods

### Adaptive Rank Truncated Product of SNP Association

One difficulty in extending the pathway enrichment analysis of genes to SNPs is the many-to-one mapping from SNPs to genes. Generally, assigning the most significant SNP to a gene might miss other informative SNPs, while assigning too many SNPs to a gene might introduce noise and decrease statistical power. Both would introduce bias into the following pathway enrichment analysis. We select the best representative subgroup of SNPs for each gene in the following way.

For each SNP, a P-value is obtained by comparing the genotype frequencies between the cases and controls using the Pearson's chi-square test with two degrees of freedom. Extending the work of Yu *et al*. [18], we use an adaptive rank truncated product method. The L P-values of the L SNPs mapped to a gene are sorted from smallest to largest: *p*_(1) _≤ *p*_(2) _≤ ... ≤ *p*_(*L*)_, with *p*_(*l*) _being the *lth *smallest *P*-value. We use  to combine the first K P-values, where K is the truncation point. Permuting the phenotypes and computing the statistic in permutated data allows us to assess the overall significance of the K SNPs. In the permutation procedure, we permute the phenotype values N times to obtain N permutated datasets. For the *nth *permutated dataset, we denote the resulting P-values as , and calculate the corresponding . Then the P-value for evaluating *W*_(*K*) _is calculated by . To maximize the association of the subset of SNPs and the trait, all possible values of K are calculated and the one with the smallest P-value is chosen. The corresponding SNPs are used to represent the gene.

To avoid genes with larger number of SNPs dominating a pathway in the following SNP set enrichment analysis, and to let the contributions by more genes be emphasized in pathway analysis, we require genes in the same pathway have the same number of representative SNPs. Therefore, for each pathway, we calculate the average number of representative SNPs of genes and re-select SNPs using this number in the given pathway.

The computation needed for selecting representative SNPs for genes involves hundreds of permutations of thousands of subjects, recalculating the test statistic in each permutation based on about half a million SNPs, and testing on multiple values of the cutoff (i.e. threshold) point K. One way to limit the computational effort is to set the upper limit K_upper _to 10 for the truncation point K. To further reduce the computational cost, we discard SNPs with large nominal P-values. On the other hand, if too few SNPs are selected, we might miss SNPs have low or moderate individual effects but jointly show a moderate or large effect. To seek a balance, we set a nominal threshold that is generous, say 0.05, i.e, only SNPs with P-values less than or equal to 0.05 will be selected. However, if none of the SNPs for a gene passes the threshold, the smallest SNP would be selected to avoid missing too many genes in pathway analysis. Both K_upper _and P-value thresholds are changeable in our software; other values can be used depending on the situation. In our experiment, we found that 10 as K_upper _and 0.05 as the P-value threshold are useful choices.

### SNP-based Pathway Enrichment Analysis

To conduct pathway analysis of SNP data from GWAS, we modified an existing gene set enrichment analysis (GSEA) algorithm [17]. The original GSEA algorithm ranks all genes by their significance of differential expression and then looks for groups of biologically relevant genes that are enriched at either the top or bottom of the ranked list. To apply this idea to SNP data, we take the N selected representative SNPs across all the genes to form the SNP list, and compute the P-values for comparing genotype frequencies between cases and controls. To measure their strength of association, we define *r_i _*= Φ^-1^(1 - *p_i_*), *i *= 1,..., *N*, where Φ^-1 ^is the quantile function for the standard normal distribution. Let *r*_(1) _≥ *r*_(2) _≥ ... ≥ *r*_(*N*) _be the sorted values from largest to smallest. A gene set sharing the same functional pathway is converted to a pathway consisting of SNPs. For a SNP-based pathway with *N_H _*SNPs, we calculate a weighted Kolmogorov-Smirnov-like running sum [22] to measure the deviation of the pathway from a set of randomly picked SNPs in the genome:

with. Here *p *is a parameter that controls the weights to ensure SNPs with higher r values tend to contribute more in the pathway level. Following the original GSEA algorithm, we set *p *= 1.

### Statistical significance evaluation

The enrichment score is expected to be high if most SNPs within a pathway are at the top of the list. We examine the statistical significance of a pathway by a permutation procedure. In each permutation, we permute (i.e. exchange) the phenotype labels, re-compute the P-values for SNPs and the corresponding enrichment score (denoted as perm_ES). Due to the size of large-scale genetic data, computational complexity would become extremely high when the number of permutations is very large. We used 1,000 permutation-cycles to generate the permutated datasets. The nominal P-value is obtained by comparing the enrichment score for the observed phenotypes with scores computed from permutated phenotypes.

Adjustment for multiple testing is applied to control false positives. When many hypotheses are tested simultaneously, the probability that at least one type I error is committed is large. One common approach for accounting for multiple testing is to control the false discovery rate (FDR) [23]. The FDR is the expected proportion of falsely rejected hypotheses out of the rejected hypotheses. One can also control the family wise error rate (FWER), which is the probability of making one or more type I errors among the family of hypothesis tests. When the number of tests is large and some of the test hypotheses are in fact false, FWER is too conservative. Since multiple pathways might be involved in a complex trait, FDR, which controls the expected proportion of false discoveries, is more suited to identifying pathways relevant to a trait. To account for multiple testing in our pathway analyses, we used a robust method to estimate the false discovery rate proposed by Pounds and Cheng [24]. The q-value is the minimum FDR at which the test is called significant. For a given significant level *α*, the point estimate of q-value (*α*) is defined as

where *FDR(t) *denotes an estimate of the proportion of tests when rejecting all null hypotheses with P-values less than or equal to the significance threshold t.

## Results

**Figure 1:**
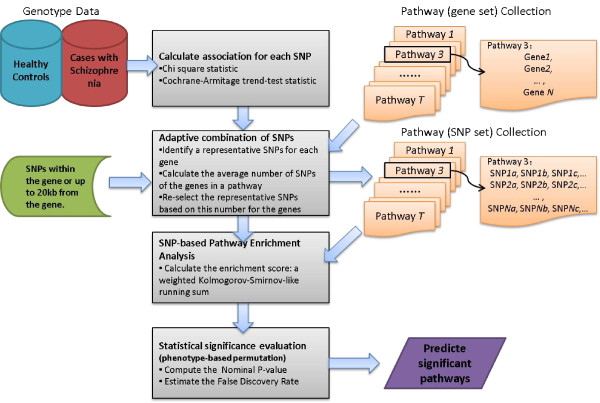
**A diagram of procedures involved in SNP set enrichment analysis (SSEA)**.

To perform SNP-based pathway enrichment analysis of GWAS data, we developed a JAVA based software package called SNP Set Enrichment Analysis (SSEA) by extending the original GSEA code. SSEA consists of four procedures as outlined in Figure 1: 1) calculating the P-value of the association of each SNP to a trait of interest, 2) selecting representative SNPs for each gene using an adaptive SNP combination method, calculating the average number of representative SNPs for genes in each pathway and reselecting SNPs for gene in each pathway, 3) ranking all selected SNPs by their P-values and testing if the SNPs from a pathway are enriched with high ranks, and 4) calculating the FDR of the discovered pathways. See Methods for details.

We applied SSEA to two large genetically distinct GWAS data sets for schizophrenia from the Genetic Association Information Network (GAIN, http://www.genome.gov/19518664) studies [25], available at the database of Genotype and Phenotype (dbGaP) [26]. The study version we reported here is phs000021.v2.p1 with general research use consent, which includes two samples; one is from the European American (EA) ancestry and the other one is from African American (AA) ancestry. Individuals in those two cohorts represent two genetically distinct populations [27,28]. However, we should note that the two data sets were collected and quality controlled in a similar way, which might affect the independence of the two data sets. Both samples were genotyped by the Affymetrix SNP array 6.0. With GAIN quality-control criteria and after removing redundant subjects, the data sets included 1172 cases and 1378 controls in EA and 921 cases and 954 controls in AA. Since Linkage Disequilibrium (LD) is an important concern for selecting representative SNPs for each gene, we used Plink http://pngu.mgh.harvard.edu/~purcell/plink/ to prune SNPs that are in strong LD (Plink uses 0.5 as the default pairwise R^2 ^threshold, Supplementary Table 3 in Additional file 1). The final data used in our study consisted of 245,216 SNPs in EA and 482,914 SNPs in AA. The SNPs were assigned to genes on the basis of being located within the gene or up to 20 kb from the gene. Most genes are associated with more than one SNP; we applied the adaptive rank truncated product of SNP association algorithm described in Method to selected representative SNPs for each gene. For pathways, we used 215 experimentally validated pathways from the KEGG database[29] (Release 55, accessed 12 September).

Application of SSEA to the two schizophrenia data sets resulted in the discovery of 22 pathways in the EA data set and 11 pathways in the AA data set with the nominal P-value less than or equal to 0.001. Using this P-value cut-off, the overall FDR is controlled within 5% for both data sets. The list of identified pathways from each sample is shown in Additional file 1 and 2, together with the related gene information.

Interestingly, the two data sets share 8 significant pathways; we used Monte Carlo simulation to assess the significance of sharing and found the P-value is less than 1.0E-6. To examine whether our method detects biologically relevant pathways or random combinations of genes, we permutated genes and generated 215 random pathways for both EA and AA data sets; Our method only detected 6 significant pathways (P <= 0.001) in EA and 3 in AA, and none of them is shared, indicating that the number of significant pathways detected by our methods is more than what expected by chance, and those significant pathways are likely to be biologically relevant. The list of the 8 replicated pathways are shown in Table 1, together with their nominal P-values, the gene set sizes and the SNP set sizes associated with each pathway, and the full list of 22 significant pathways in EA and 11 significant pathways in AA are shown in Additional file 2.

**Table 1:** Eight significant pathways (P <= 0.001) discovered in both European-American ancestry and African-American ancestry data sets of schizophrenia

PATHWAYS		European Ancestry (EA)			African Ancestry (AA)		
		
		Nom P	GENE SIZE	SNP SIZE	Nom P	GENE SIZE	SNP SIZE
HSA04720	Long-term potentiation	<0.001	67	135	0.001	67	66

HSA04270	Vascular smooth muscle contraction	<0.001	107	215	<0.001	107	105

HSA05412	Arrhythmogenic right ventricular cardiomyopathy (ARVC)	<0.001	72	144	<0.001	72	135

HSA04020	Calcium signaling pathway	<0.001	168	337	<0.001	174	165

HSA04360	Axon guidance	<0.001	122	245	<0.001	126	120

HSA04080	Neuroactive ligand-receptor interaction	<0.001	256	509	<0.001	266	248

HSA04510	Focal adhesion	<0.001	186	378	<0.001	191	186

HSA04730	Long-term depression	< 0.001	68	134	0.001	68	129

Schizophrenia [MIM 181500] is a complex brain disorder characterized by disturbances in multiple domains of brain function, including cognitive, emotional, and perceptual processes [30]. Evidence for schizophrenia as a neurodevelopment disorder began more than 30 years ago [31] and has been accepted commonly [32]. It is intriguing to note that the 8 pathways discovered by SSEA in both the EA and AA samples included 4 pathways important for neurodevelopment and neuronal functioning, such as axon guidance pathway, neuroactive ligand-receptor interaction pathway, long-term depression pathway and long-term potentiation pathway. Axon guidance pathway and neuroactive ligand-receptor interaction pathway are directly related to neuroplasticity and neuropathology, and thus are important to the genetic mechanism of schizophrenia [33]. Long-term depression pathway and long-term potentiation pathway were reported to be important for synaptic plasticity development and related to schizophrenia [34,35]. Besides, axon guidance pathway, long-term depression pathway and long-term potentiation pathway were reported in a recent study where pathways were overrepresented by genes disrupted by copy number variants in schizophrenia cases [36]. Genes in the focal adhesion pathway are principally involved in the biological processes for synaptic transmission and cell adhesion [37]. In addition, arrhythmogenic right ventricular cardiomyopathy (ARVC) pathway is related to cardiovascular disease, which supports the previous study that patients with schizophrenia had higher rates of cardiovascular disease and mortality compared with the general population [38,39]. We took a further investigation of gene intersections in the remaining two non-schizophrenia specific pathways. We found the calcium signalling pathway shared 40 (24%) genes with long-term potentiation pathway, while vascular smooth muscle contraction shared 40(37%) genes with long-term depression pathway; the same genes implicated in different pathways might be a reason for their enrichment in our study.

As a comparison, we also applied another two methods, smallest P-value and PLINK set-based tests [40], to the two GAIN data sets. The smallest P-value method, which only used the SNP with the smallest P-value to represent a gene, detected 6 and 2 significant pathways in EA and AA data sets, respectively; only one was shared by both data sets. This showed our method could improve the power of detecting causal pathways by using multiple SNPs to represent a gene. For the set-based test method (with parameters --set-p 0.05, --set-r2 0.5, the same as that in our method), 3 significant pathways were detected in EA, and 2 in AA, but none was shared between the two data sets. One reason for the loss of power of these two methods might be their favouring of pathways containing large numbers of genes and genes with large number of SNPs, as larger pathways are expected to show more significant genes or SNPs just by chance. We checked several potential factors that might affect pathway significance: pathway size, gene size, total *bp *content, and average content. We found these factors are uncorrelated with pathway significance (Supplementary Figure 1a,b,c,d in Additional file 1), confirming that using multiple representative SNPs per gene and permutations are able to reduce the bias introduced by gene and pathway sizes.

Relaxing the nominal P-value cut-off to 0.01, with FDR q-value controlled within 10%, resulted in 40 significant pathways detection in EA data set, and 27 significant pathways in AA data set. Among them, 17 pathways are shared (Monte Carlo simulation P-value for sharing is less than 1.0e-6). The full list of 17 shared pathways is shown in Table 2.

**Table 2:** Seventeen significant pathways (P <= 0.01) discovered in both European-American and African-American ancestry samples of schizophrenia

PATHWAYS		European Ancestry (EA)			African Ancestry (AA)		
		
		Nom P	GENE SIZE	SNP SIZE	Nom P	GENE SIZE	SNP SIZE
HSA04720	Long-term potentiation	0.001	67	66	<0.001	69	135

HSA04270	Vascular smooth muscle contraction	<0.001	107	105	<0.001	113	215

HSA05412	Arrhythmogenic right ventricular cardiomyopathy (ARVC)	<0.001	72	135	<0.001	74	144

HSA04020	Calcium signaling pathway	<0.001	168	165	<0.001	174	337

HSA04972	Pancreatic secretion	0.008	93	91	<0.001	94	184

HSA04360	Axon guidance	<0.001	122	120	<0.001	126	245

HSA04080	Neuroactive ligand-receptor interaction	<0.001	256	248	<0.001	266	509

HSA04510	Focal adhesion	<0.001	186	186	<0.001	191	378

HSA04730	Long-term depression	<0.001	68	129	0.001	68	134

HSA00330	Arginine and proline metabolism	0.001	47	47	0.002	52	102

HSA04970	Salivary secretion	0.003	80	151	0.002	86	166

HSA05146	Amoebiasis	0.009	100	99	0.003	103	199

HSA05414	Dilated cardiomyopathy	<0.001	88	86	0.005	90	173

HSA04070	Phosphatidylinositol signaling system	0.002	75	74	0.006	77	150

HSA04512	ECM-receptor interaction	0.001	81	81	0.007	82	161

HSA04260	Cardiac muscle contraction	0.009	63	61	0.009	67	128

HSA04540	Gap junction	0.003	80	80	0.009	85	165

## Discussion

The traditional strategy for GWAS studies tests one SNP at a time. Although widely used, single-SNP GWAS analysis does not have adequate power to detect SNPs that have marginally weak, but jointly strong genetic effects. Jointly analyzing SNPs within the same biological pathway simultaneously complements the single-SNP analysis and can reveal new insights to the understanding of complex human traits. Our SNP set enrichment analysis operates on representative SNPs of genes and then combines the effects of SNPs within the same pathway by a weighted Kolmogorov-Smirnov running sum statistic test [22]. This strategy has the potential to increase the chance of identifying genetic variants that that individually have a modest risk.

Compared to gene set enrichment analysis, the SNP set enrichment analysis is a much larger scale and is more computationally challenging. Several pathway-based methods have recently been developed to analyse GWAS [13,20,41-44]. In general, these methods can be classified into two categories, depending upon how representative SNPs for each gene are chosen: one selects the most significant SNP per gene, and the other selects all SNPs located within a gene [20]. Both approaches have limitations. Using all available SNPs per gene not only poses computational challenges, but also introduces significant amounts of noise into the analysis. Using the most significant SNP per gene might miss SNPs with moderate strength individually but strong effects jointly, and in addition it introduces biases of favouring large extensive pathways and genes with greater numbers of SNPs. The SSEA method we proposed uses an adaptive approach to choose SNPs in each gene, and can eliminate the limitations of other strategies.

It is also worthy to point out that the number of selected SNPs varies between genes. This is because we used permutations to decide both the number and the set of SNPs to represent each gene. The permutation of phenotypes and recalculation of statistical values for about half a million SNPs and thousands of subjects is computationally expensive. To seek a balance between the computational complexity and not losing too much information from SNPs, we set a nominal significance threshold chose only SNPs with smaller P-value for pathway analysis. To further reduce computation, we recommend using an upper limit for the number of representative SNPs for each gene.

Our method has a critical assumption. In combing P-values of SNPs in a gene we assume that the P-values are independent, although in reality some SNPs in a gene are in linkage disequilibrium (LD). When comparing the results with and without removing SNPs in strong LD, we found there is no big difference between them. However, a future direction is to relax this assumption and develop a SNP selection method that explicitly takes the LD patterns into account rather than remove SNPs in LD. It is interesting to note that Peng *et al*. [15] also found that ignoring LD could actually lead to better results than methods with very conservative multiple testing corrections. The permutation test we consider might partially alleviate the effect due to LD.

A critical component for the success of the pathway-based analysis is the availability of a comprehensive collection of relevant gene sets related to the disease/genetic trait of interest. Current understanding of gene functions and pathways is still very limited. This is especially the case for neuropsychiatric diseases, as most of the gene sets currently available were generated based on experiments done on tumor cell lines. As a consequence, we have only limited knowledge regarding the pathways involved in brain development, and normal and pathological activities. In this regard, the pathways discovered by SSEA for schizophrenia are likely to be substantially incomplete. We expect the performance would improve as better and more comprehensive disease-related pathways become available. A future challenge is to curate pathways and gene sets in a disease specific way, possibly by taking advantage of the high-throughput functional genomics tools.

## Conclusion

In summary, we have developed a new SNP-based method, called SNP Set Enrichment Analysis (SSEA), for pathway analysis of GWAS data. SSEA selects a multiple and varying number of SNPs to represent each gene using an adaptive truncated product statistic. The selected SNPs are then ranked and enrichment of pathways is tested using a weighted Kolmogorov-Smirnov test. We tested SSEA in two genetically distinct GWAS studies of schizophrenia with large samples, and discovered 22 significant pathways in the European-American sample and 11 significant pathways in the African-American sample. Eight important pathways were found in both distinct samples providing support for our method.

The SSEA method is coded in a JAVA software package with a user-friendly interface. The software is freely available at http://cbcl.ics.uci.edu/SSEA/.

## Authors' contributions

Designed the experiments: WL, SGP, YZ and XX; Performed the experiments: WL; Wrote the paper: WL, FM, YZ, and XX; All authors contributed to the analysis, and approved the paper.

## Supplementary Material

Additional file 1 **Supplementary tables and figures on genes and pathways**. Supplementary tables and figures on genes and pathways discovered by SSEA. Click here for file

Additional file 2 **Detailed information on replicated pathways**. A excel file containing detailed information on replicated pathways. Click here for file
