# Supplementary material for: SNP-based pathway enrichment analysis for genome-wide association studies
Source: BMC Bioinformatics. 2011 Apr 15;12:99. doi: 10.1186/1471-2105-12-99 (PMC3102637; doi:10.1186/1471-2105-12-99)
Supplement: Additional file 1 — Supplementary tables and figures on genes and pathways. Supplementary tables and figures on genes and pathways discovered by SSEA. [file 1471-2105-12-99-S1.PDF]

Supplementary Table 1

Genes overlapping between 8 significant pathways in EA data set

	HSA04360	HSA04730	HSA04720	HSA04080	HSA04020	HSA04270	HSA05412	HSA04510
HSA04360	126	7	11	0	7	7	5	19
HSA04730	7	68	24	7	18	40	0	10
HSA04720	11	24	67	9	40	32	1	14
HSA04080	0	7	9	256	59	10	0	0
HSA04020	7	18	40	59	168	43	7	10
HSA04270	7	40	32	10	43	107	4	18
HSA05412	5	0	1	0	7	4	72	27
HSA04510	19	10	14	0	10	18	27	186

Genes overlapping between 8 significant pathways in AA data set

	HSA04360	HSA04730	HSA04720	HSA04080	HSA04020	HSA04270	HSA05412	HSA04510
HSA04360	126	7	11	0	7	5	1	20
HSA04730	7	69	24	7	18	40	0	10
HSA04720	11	24	67	9	42	34	1	14
HSA04080	0	7	9	256	62	10	0	0
HSA04020	5	18	42	62	168	45	7	10
HSA04270	7	40	34	10	45	107	4	18
HSA05412	1	0	1	0	7	4	72	27
HSA04510	20	10	14	0	10	18	27	186

Supplementary Table 2

Number of significant pathways detected under different LD-pruning criteria in EA and AA data set.

	Original SNP data	After remove SNPs with r2 >0.9	After remove SNPs with r2 >0.5	Shared pathways under different LD pruning criteria
European-American(EA)	16	18	22	13
African-American (AA)	9	8	11	6
Replicated pathways in EA and AA	6	6	8	5

Supplementary Table 3

A) European-American (EA) sample:

Not pruned		Pruned with r2=0.9		Pruned with r2=0.5	
Pathway	p	pathway	p	pathway	p
HSA00360	<0.001	HSA00330	<0.001	HSA04510	<0.001
HSA00330	<0.001	HSA00360	<0.001	HSA05412	<0.001
HSA04360	<0.001	HSA04914	<0.001	HSA04914	<0.001
HSA04514	<0.001	HSA04080	<0.001	HSA04270	<0.001
HSA05412	<0.001	HSA04360	<0.001	HSA04512	<0.001
HSA04720	<0.001	HSA04010	<0.001	HSA05414	<0.001
HSA04914	<0.001	HSA05412	<0.001	HSA05410	<0.001
HSA05414	<0.001	HSA04145	<0.001	HSA04514	<0.001
HSA04510	<0.001	HSA04020	<0.001	HSA04020	<0.001
HSA04020	<0.001	HSA04514	<0.001	HSA04010	<0.001
HSA04512	<0.001	HSA04510	<0.001	HSA05200	<0.001
HSA04080	<0.001	HSA05200	<0.001	HSA04810	<0.001
HSA04912	0.001	HSA00340	0.001	HSA04080	<0.001
HSA04270	0.001	HSA00350	0.001	HSA04145	<0.001
HSA04730	0.001	HSA04720	0.001	HSA05213	<0.001
HSA04010	0.001	HSA04912	0.001	HSA04360	<0.001
		HSA04730	0.001	HSA00330	<0.001
		HSA04512	0.001	HSA04912	<0.001
				HSA04730	0.001
				HSA04720	0.001
				HSA00280	0.001
				HSA00603	0.001

B) African-American (AA) sample

Not pruned		Pruned with r2=0.9		Pruned with r2=0.5	
pathway	p	pathway	p	pathway	p
HSA04720	<0.001	HSA04020	<0.001	HSA04720	<0.001
HSA04020	<0.001	HSA04720	<0.001	HSA04270	<0.001
HSA00230	<0.001	HSA04080	<0.001	HSA05412	<0.001
HSA04360	<0.001	HSA04510	<0.001	HSA04020	<0.001
HSA00260	0.001	HSA00512	0.001	HSA04972	<0.001
HSA05412	0.001	HSA04360	0.001	HSA04360	<0.001
HSA04080	0.001	HSA00230	0.001	HSA04080	<0.001
HSA04270	0.001	HSA05412	0.001	HSA04510	<0.001
HSA00512	0.001			HSA00512	0.001
				HSA04971	0.001
				HSA04730	0.001

We used Linkage disequilibrium (LD) based SNP pruning provided by PLINK to remove SNPs in strong LD. We tried two different pair-wise r2 thresholds (0.9 and 0.5). After implementing our method to the EA original, EA 0.9LD-pruned and EA 0.5LD-pruned data sets, we detected 16, 18 and 22 significant pathways with p<=0.001, respectively, and 13 of them are replicated in all three data sets. While for AA original, AA 0.9 LD-pruned and AA 0.5 LD-pruned data sets, we detected 9, 8, 11 significant pathways with p<=0.001, respectively, and 6 of them are replicated in all three data sets.

Supplementary Figure 1

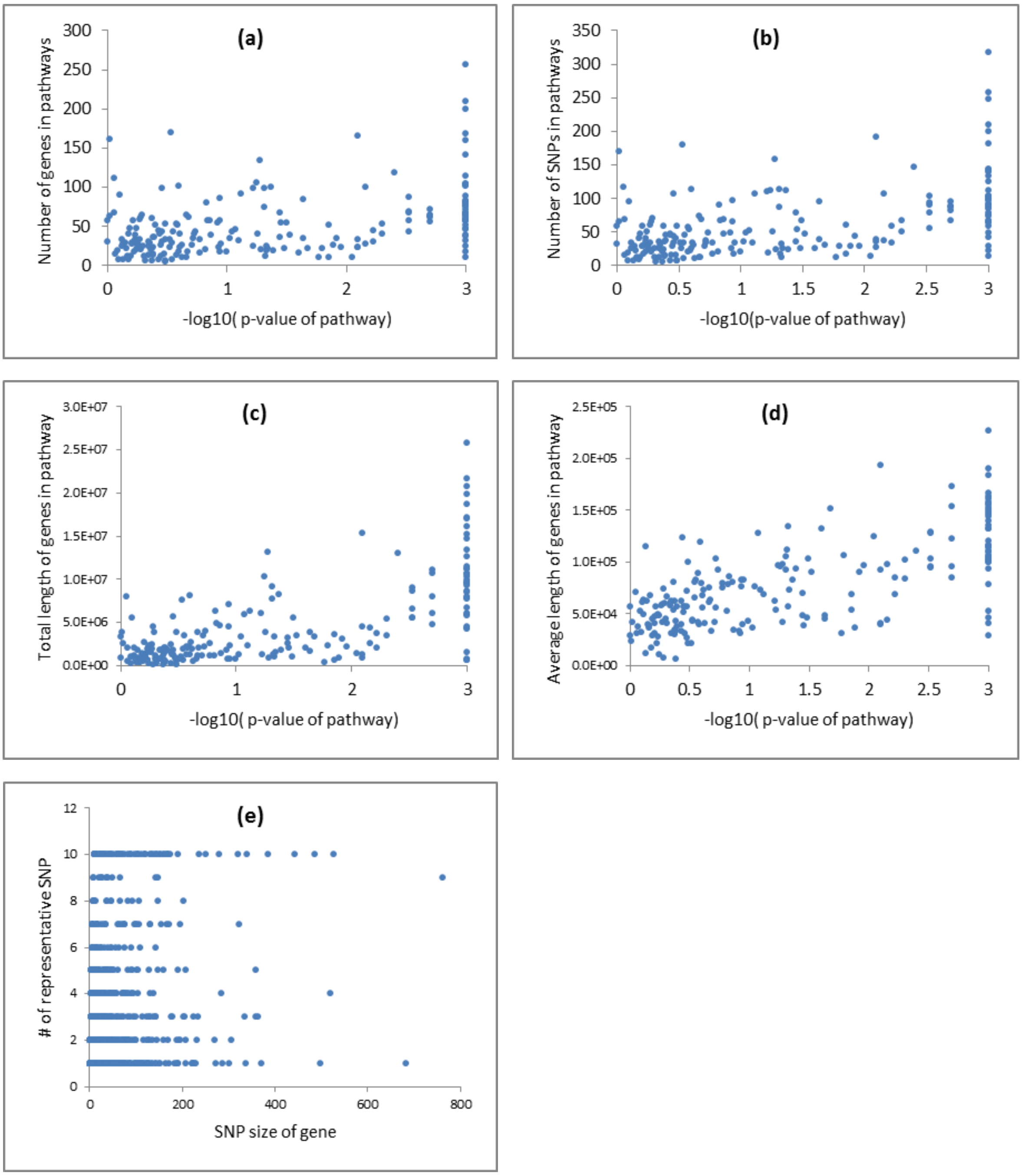

Supplementary Figure 1 the significance of pathway(-log10(P-value)) in EA versus (a) the number of genes in pathways, (b) the number of significant SNPs in pathways, (c) Total length (*bp*) of genes in pathways, (d) average length (*bp*) of genes in pathways.(e)the number of representative SNPs selected verse the number of SNPs belongs to a gene.
